# Supplementary material for: Exploring the Potential of Malvidin and Echiodinin as Probable Antileishmanial Agents Through In Silico Analysis and In Vitro Efficacy
Source: Molecules. 2025 Jan 4;30(1):173. doi: 10.3390/molecules30010173 (PMC11722285; doi:10.3390/molecules30010173)
Supplement: Supplementary file 1 [file molecules-30-00173-s001.zip › molecules-3357460-supplementary.pdf]

Supplementary material

Exploring the Potential of Malvidin and Echiodinin as Probable Antileishmanial Agents Through In Silico Analysis and In Vitro Efficacy

Luis Daniel Goyzueta-Mamani [1], Daniela Pagliara Lage [2], Haruna Luz Barazorda-Ccahuana [1], Margot Paco-Chipana [1], Mayron Antonio Candia-Puma [1,3], Gonzalo Davila-Del-Carpio [3], Alexsandro Sobreira Galdino [4,5], Ricardo Andrez Machado-de-Avila [6], Rodolfo Cordeiro Giunchetti [7,8], Edward L. D'Antonio [9], Eduardo Antonio Ferraz Coelho [2] and Miguel Angel Chávez-Fumagalli [1,*]

1 Computational Biology and Chemistry Research Group, Vicerrectorado de Investigación, Universidad Católica de Santa María, Arequipa 04000, Peru; lgoyzueta@ucsm.edu.pe (L.D.G.-M.); hbarazorda@ucsm.edu.pe (H.L.B.-C.); 74252022@ucsm.edu.pe (M.P.-C.); mcandia@ucsm.edu.pe (M.A.C.-P.)
2 Programa de Pós-Graduação em Ciências da Saúde: Infectologia e Medicina Tropical, Faculdade de Medicina, Universidade Federal de Minas Gerais, Belo Horizonte 31270-901, Brazil; dpagliarara@icb.ufmg.br (D.P.L.); eduardoferrazcoelho@yahoo.com.br (E.A.F.C.)
3 Facultad de Ciencias Farmacéuticas, Bioquímicas y Biotecnológicas, Universidad Católica de Santa María, Arequipa 04000, Peru; gdavilad@ucsm.edu.pe
4 Laboratório de Biotecnologia de Microrganismos, Universidade Federal São João Del-Rei, Divinópolis 35501-296, Brazil; asgaldino@ufsj.edu.br
5 Instituto Nacional de Ciência e Tecnologia em Biotecnologia Industrial (INCT-BI), Distrito Federal, Brasilia 70070-010, Brazil
6 Programa de Pós-Graduação em Ciências da Saúde, Universidade do Extremo Sul Catarinense, Criciúma 88806-000, Brazil; r_andrez@unesc.net
7 Laboratório de Biologia das Interações Celulares, Instituto de Ciências Biológicas, Universidade Federal de Minas Gerais, Belo Horizonte 31270-901, Brazil; giunchetti@icb.ufmg.br
8 Instituto Nacional de Ciência e Tecnologia de Doenças Tropicais (INCT-DT), Salvador 40110-160, Brazil
9 Department of Natural Sciences, University of South Carolina Beaufort, 1 University Boulevard, Bluffton, SC 29909, USA; edantonio@uscb.edu
* Correspondence: mchavezf@ucsm.edu.pe

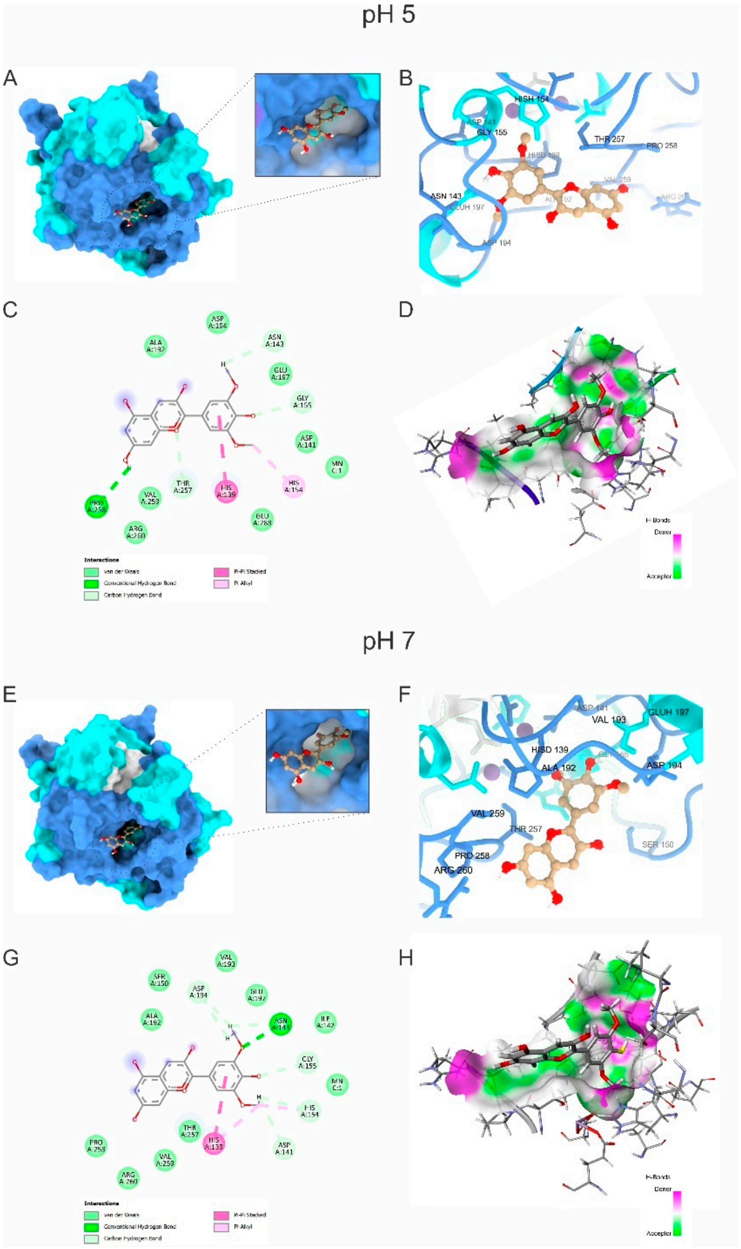

Figure S1. Docking of Malvidin with arginase protein (ARG) from *Leishmania amazonensis*. Panels A, B, C, and D depict the 3D surface representation, 3D structure of the binding mode and molecular interactions of the hit ligands, 2D view of interaction types, and H-bond interacting surface at pH 5, respectively. Panels E, F, G, and H show the corresponding visualizations at pH 7.

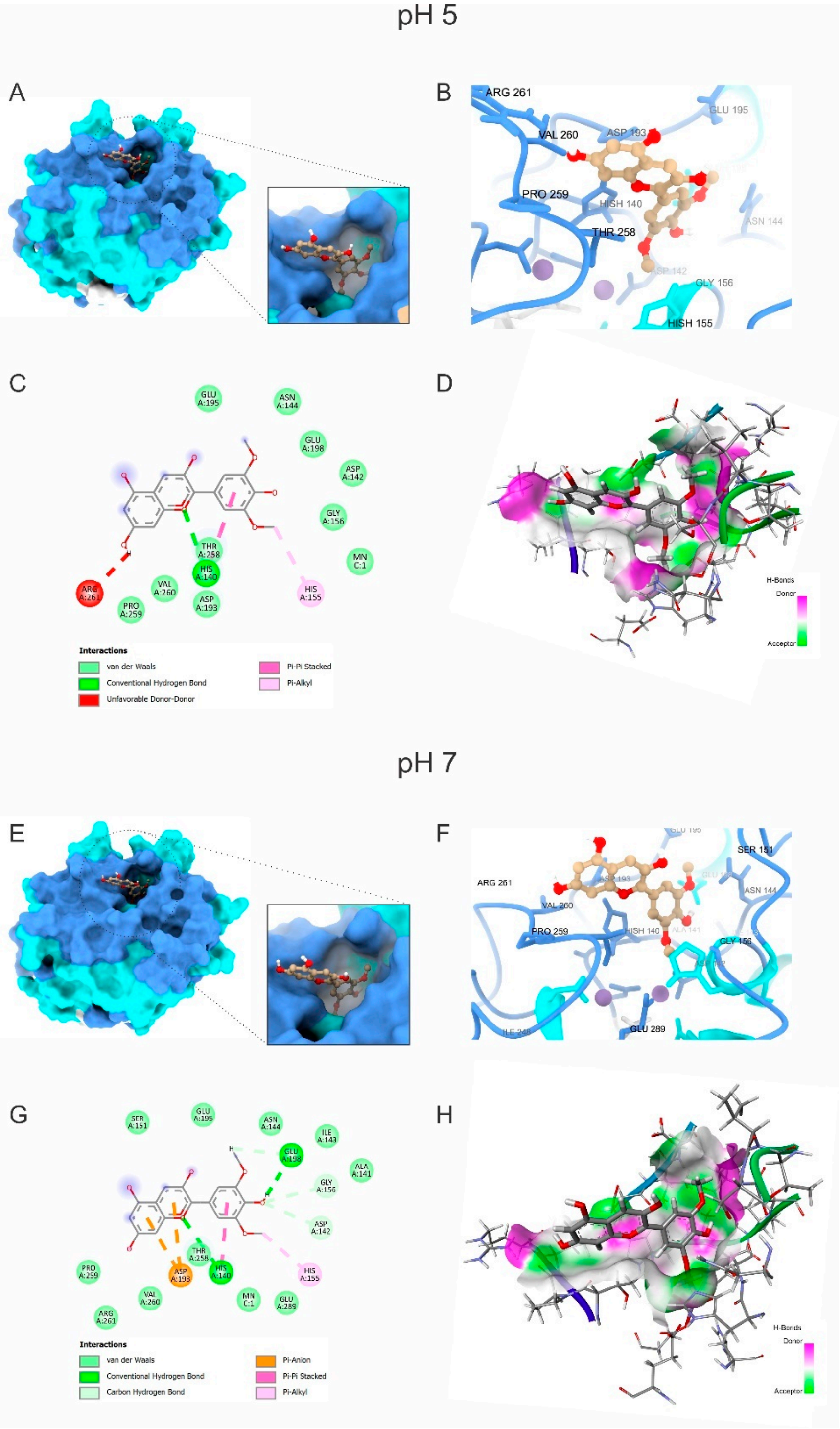

Figure S2. Docking of Malvidin with arginase protein (ARG) from *Leishmania braziliensis*. Panels A, B, C, and D depict the 3D surface representation, 3D structure of the binding mode and molecular interactions of the hit ligands, 2D view of interaction types, and H-bond interacting surface at pH 5, respectively. Panels E, F, G, and H show the corresponding visualizations at pH 7.

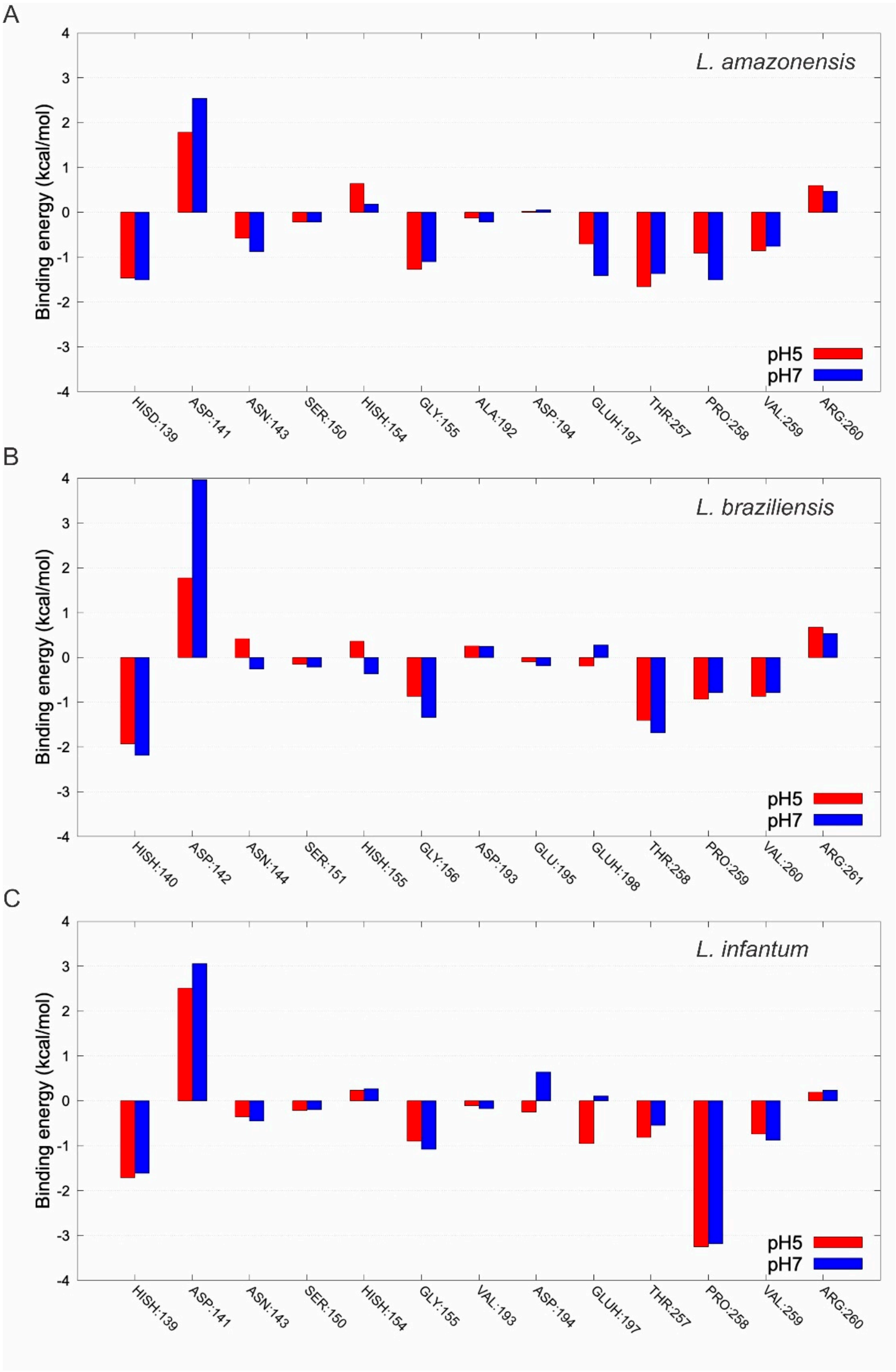

Figura S3. Decomposition of free energy per residue using the MM/GBSA method, showing the highest energy contribution from the ARG hot spot in interaction with Malvidin.
